# Supplementary material for: Discovery of Unconventional Kinetochores in Kinetoplastids
Source: Cell. 2014 Mar 13;156(6):1247–58. doi: 10.1016/j.cell.2014.01.049 (PMC3978658; doi:10.1016/j.cell.2014.01.049)
Supplement: Table S5. ChIP-Seq Statistics, Related to Figure 2 [file mmc5.pdf]

Table S5. ChIP-Seq Statistics, Related to Figure 2

| | **YFP-KKT2** | **YFP-KKT3** | **YFP-H3v** |
|---|---|---|---|
| **Total reads** | | | |
| Input | 11,738,554 | 16,194,294 | 15,286,210 |
| ChIP | 9,520,838 | 12,760,681 | 14,948,206 |
| **Mapped reads** | | | |
| Input | 10,349,757 (88.17%) | 14,085,156 (86.98%) | 13,484,877 (88.22%) |
| ChIP | 8,442,798 (88.68%) | 10,644,613 (83.42%) | 12,923,566 (86.46%) |
