# Supplementary material for: Discovery of Unconventional Kinetochores in Kinetoplastids
Source: Cell. 2014 Mar 13;156(6):1247–58. doi: 10.1016/j.cell.2014.01.049 (PMC3978658; doi:10.1016/j.cell.2014.01.049)
Supplement: Document S1. Article plus Supplemental Information [file mmc6.pdf]

Cell

Discovery of Unconventional Kinetochores in Kinetoplastids

Bungo Akiyoshi[1,2,*] and Keith Gull[1]
[1]Sir William Dunn School of Pathology, University of Oxford, Oxford OX1 3RE, UK
[2]Department of Biochemistry, University of Oxford, Oxford OX1 3QU, UK
*Correspondence: bungo.akiyoshi@bioch.ox.ac.uk
http://dx.doi.org/10.1016/j.cell.2014.01.049
This is an open-access article distributed under the terms of the Creative Commons Attribution License, which permits unrestricted use, distribution, and reproduction in any medium, provided the original author and source are credited.

SUMMARY

The kinetochore is the macromolecular protein complex that directs chromosome segregation in eukaryotes. It has been widely assumed that the core kinetochore consists of proteins that are common to all eukaryotes. However, no conventional kinetochore components have been identified in any kinetoplastid genome, thus challenging this assumption of universality. Here, we report the identification of 19 kinetochore proteins (KKT1–19) in *Trypanosoma brucei*. The majority is conserved among kinetoplastids, but none of them has detectable homology to conventional kinetochore proteins. These proteins instead have a variety of features not found in conventional kinetochore proteins. We propose that kinetoplastids build kinetochores using a distinct set of proteins. These findings provide important insights into the longstanding problem of the position of the root of the eukaryotic tree of life.

INTRODUCTION

Faithful transmission of genetic material is essential for the survival of all organisms. Eukaryotic chromosome segregation is driven by the kinetochore, a macromolecular protein complex that assembles onto centromeric DNA and captures spindle microtubules to govern the movement of chromosomes (Cheeseman and Desai, 2008; Santaguida and Musacchio, 2009). Kinetochores consist of more than 40 different components even in the simple yeast kinetochore (Biggins, 2013) and are recognized as one of the most complex structures in the cell. A hallmark of eukaryotic kinetochores is the centromere-specific histone H3 variant (CENP-A), which specifies the site of kinetochore assembly by creating a specialized chromatin environment (Hori and Fukagawa, 2012; Maddox et al., 2012; Westhorpe and Straight, 2013). Putative CENP-A homologs can be identified in nearly all sequenced eukaryotes (Talbert et al., 2009), suggesting that most eukaryotes utilize CENP-A to assemble kinetochores. However, notable exceptions are found in the kinetoplastid species, a group of unicellular flagellated eukaryotes, including parasitic Trypanosomatida (e.g., *Trypanosoma brucei*, *Trypanosoma cruzi*, and *Leishmania* species) and free-living Bodonida (e.g., *Bodo saltans*). Their genome sequences have so far failed to reveal any CENP-A homolog (Lowell and Cross, 2004; Berriman et al., 2005), suggesting that kinetoplastids assemble kinetochores without CENP-A, possibly using a distinct set of proteins. Consistent with this possibility, bioinformatic analyses have failed to detect any conventional kinetochore protein homolog in kinetoplastids, whereas at least a fraction of kinetochore components can be readily identified in other diverse eukaryotes (Meraldi et al., 2006; Westermann and Schleiffer, 2013) (Table S1 available online). By comparison, kinetoplastids possess the CDK/cyclin system, cohesin complex, separase, condensin complex, Aurora B, the anaphase promoting complex (APC/C), and proteasomes, suggesting that the most basic cell-cycle machinery is conserved in these distant eukaryotes (Akiyoshi and Gull, 2013).

T. brucei is the causative agent of devastating African sleeping sickness in humans and nagana in livestock. In addition to 11 homologous pairs of large chromosomes (also called megabase chromosomes, 1–6 Mb in size), *T. brucei* possesses ∼100 small chromosomes (minichromosomes, 50–150 kb; intermediate chromosomes, 200–700 kb) (Daniels et al., 2010; Ersfeld, 2011). Previous studies suggest that megabase chromosomes contain regional centromeres, whereas intermediate or minichromosomes may not contain canonical centromeres (Obado et al., 2007). The core of minichromosomes consists of 177 bp repeats and is constructed in a palindromic manner (Wickstead et al., 2004). Although minichromosomes do not possess housekeeping genes, they are crucial for increasing the capacity of antigenic variation (Sloof et al., 1983) and individual minichromosomes appear to segregate faithfully at each cell division (Wickstead et al., 2003). *T. brucei* undergoes a closed mitosis and forms a mitotic spindle within the nucleus (Ogbadoyi et al., 2000), and segregation of both megabase chromosomes and minichromosomes depends on spindle microtubules (Ersfeld and Gull, 1997). Ultrastructural studies have detected kinetochore-like electron-dense plaques that appear to form end-on attachments to spindle microtubules in mitotic cells (Ogbadoyi et al., 2000). Through blocking the accurate segregation of these chromosomes, cell growth or immune evasion could be inhibited. Understanding the underlying molecular mechanism is therefore critical to developing treatment strategies against kinetoplastid

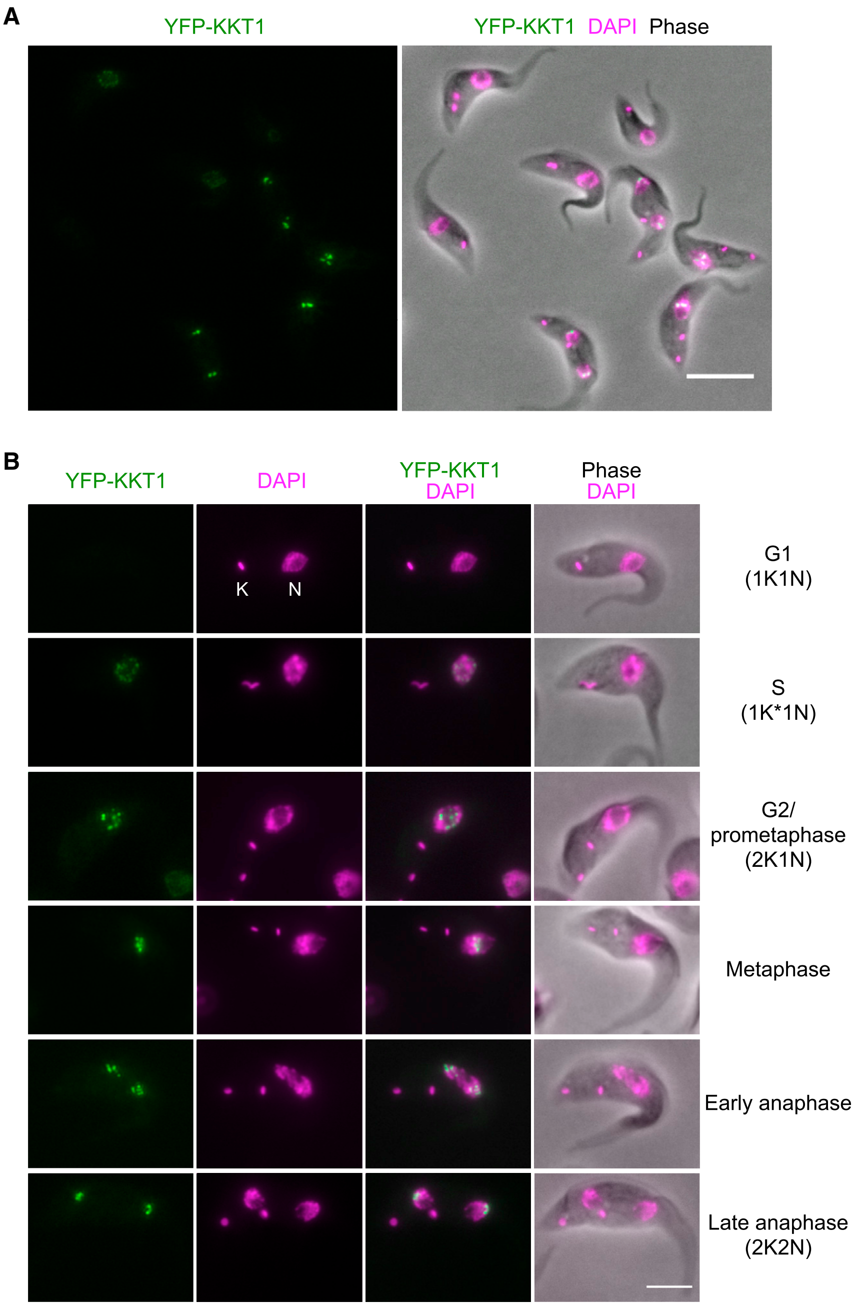

Figure 1. Identification of KKT1

(A) A wide field of view of procyclic form trypanosome cells expressing YFP-KKT1. Bar, 10 μm.

(B) Examples of cells at indicated cell-cycle stages. K and N stand for the kinetoplast and nucleus, respectively. K* denotes an elongated kinetoplast. Bar, 5 μm.

See also Figure S1 and Tables S1 and S2.

diseases. Furthermore, there is a great interest in understanding how kinetochores can be assembled in the absence of a CENP-A homolog in kinetoplastids. Identification of kinetochore proteins is an essential step toward both of these goals. Here, we describe the identification of 19 kinetochore proteins in *T. brucei*.

RESULTS

Identification of KKT1 in *T. brucei*

T. brucei possesses two DNA-containing organelles, the nucleus and the kinetoplast. The former contains nuclear DNA, whereas the latter contains a cluster of mitochondrial DNA. These organelles have distinct replication and segregation timings and serve as good cell-cycle markers (Woodward and Gull, 1990; Siegel et al., 2008). To identify proteins that are relevant for mitosis, we carried out a yellow fluorescent protein (YFP)-tagging screen to examine the localization of uncharacterized proteins whose transcript levels are upregulated later during the cell cycle (Archer et al., 2011). This screen identified a protein (ORF Tb927.10.6330) that has a localization pattern characteristic of kinetochore proteins (Figure 1A). There is little YFP signal in G1, and dots appear in the nucleus around S phase, align at the center of the nucleus in metaphase, and then move to opposite poles and localize near the leading edge of separating chromosomes during anaphase (Figure 1B). The protein is well conserved among kinetoplastids (Table 1), so we named it KKT1 for *k*inetoplastid *k*inetochore protein 1.

Identification of KKT2–19

To identify more kinetochore proteins, we affinity purified a YFP-tagged version of KKT1 (Figure S1) and identified the copurifying proteins by mass spectrometry (MS) (Table S2). Twelve uncharacterized proteins were identified that copurified with KKT1 in an apparently specific manner. We tagged these proteins with YFP and found that they all have kinetochore-like localization patterns (see below). We therefore named them KKT2–13. We then looked for more kinetochore proteins by the affinity purification/MS of YFP-tagged versions of these 12 proteins (Figure S1 and Table S2), followed by the YFP-tagging of candidate proteins, which led to the identification of six additional kinetochore proteins (KKT14–19). Affinity purification/MS of these six proteins (Figure S1 and Table S2) failed to identify any more kinetochore proteins, indicating that the approach had reached saturation. Although there may be more kinetochore proteins still unidentified, we began to characterize the 19 KKT proteins identified from this methodology.

Table 1. KKT Proteins Are Highly Conserved among Kinetoplastid Species

Name	*T. brucei*	*T. cruzi*	*T. congolense*	*T. vivax*	*L. mexicana*	*L. braziliensis*	*Bodo saltans*
KKT1	Tb927.10.6330	TcCLB.507641.190	TcIL3000.10.5400	TvY486_1006290[a]	LmxM.36.1900	LbrM.35.2090	BS71780
KKT2	Tb927.11.10520	TcCLB.510285.70	TcIL3000.11.11110	TvY486_1111400	LmxM.36.5350	LbrM.35.5600	BS50690
KKT3	Tb927.9.10920	TcCLB.508461.230	TcIL3000.9.4440	TvY486_0904950[a]	LmxM.34.4050	LbrM.34.4040	BS05255
KKT4	Tb927.8.3680	TcCLB.511575.70	TcIL3000.0.43630	TvY486_0803080[a]	LmxM.10.0300	LbrM.10.0320	BS42920
KKT5	Tb927.7.4850			TvY486_0704920	LmxM.06.0200	LbrM.06.0180	BS78350
KKT6	Tb927.6.1210	TcCLB.507603.70	TcIL3000.0.27570	TvY486_0600720	LmxM.12.0080	LbrM.12.0110	BS31505
KKT7	Tb927.11.1030	TcCLB.506925.490	TcIL3000.11.950	TvY486_1100920	LmxM.27.0430	LbrM.27.0520	BS46505
KKT8	Tb927.4.5110	TcCLB.510593.40	TcIL3000.8.7400	TvY486_0806790	LmxM.30.2750	LbrM.31.3100	BS23830
KKT9	Tb927.8.1150	TcCLB.506401.160	TcIL3000.0.31110	TvY486_0800590[a]	LmxM.02.0610	LbrM.02.0590	BS91660
KKT10	Tb927.11.12410	TcCLB.511127.320	TcIL3000.0.20360	TvY486_1113350	LmxM.09.0400	LbrM.09.0410[a]	BS75410
KKT11	Tb927.7.2110	TcCLB.506683.30	TcIL3000.7.2460	TvY486_0701840	LmxM.22.0120	LbrM.22.0120	BS26590
KKT12	Tb927.8.1680	TcCLB.505071.50	TcIL3000.8.1520	TvY486_0801090	LmxM.24.1400	LbrM.24.1480	BS37635
KKT13	Tb927.7.4860	TcCLB.507817.30	TcIL3000.7.4030	TvY486_0704930	LmxM.06.0210	LbrM.06.0190	BS78345
KKT14	Tb927.10.7240	TcCLB.509537.40	TcIL3000.10.6210	TvY486_1007090[a]	LmxM.36.2800	LbrM.35.3020	BS67465
KKT15	Tb927.6.3760	TcCLB.507029.50		TvY486_0603240	LmxM.29.2520	LbrM.30.2470	BS89620
KKT16	Tb927.11.1000	TcCLB.506925.460	TcIL3000.11.910	TvY486_1100870	LmxM.27.0400	LbrM.27.0490	
KKT17	Tb927.3.2330	TcCLB.508479.240	TcIL3000.3.1390	TvY486_0301690	LmxM.25.2220	LbrM.25.1800	BS79390
KKT18	Tb927.9.3800	TcCLB.511577.160	TcIL3000.9.1240	TvY486_0901270	LmxM.01.0350	LbrM.01.0400	BS26765
KKT19	Tb927.11.12420	TcCLB.511127.330	TcIL3000.11.13030		LmxM.09.0410	LbrM.09.0420	BS75425

[a]Gene fragment.

KKT Proteins Are Conserved in Kinetoplastids

The majority of KKT proteins appear to be well conserved among kinetoplastids (Table 1). However, homology search programs using position-specific iterated (PSI)-BLAST (Altschul et al., 1997) or hidden Markov models (Eddy, 1998) failed to identify homologous proteins in other organisms except for proteins that contain shared conserved domains (see below). Furthermore, we could not find any significant homology between KKT proteins and conventional kinetochore proteins. These results raise the possibility that the KKT proteins constitute an unconventional kinetochore unique to kinetoplastids, which is in line with the absence of CENP-A. Interestingly, conservation of KKT proteins in those kinetoplastids that do not possess intermediate or minichromosomes (e.g., *T. cruzi* and *Leishmania*) suggests that they are likely involved in the segregation of megabase-type chromosomes in kinetoplastids.

KKT Proteins Are Enriched at Centromeres

We first wanted to verify that KKT proteins indeed localize at the centromere of megabase chromosomes, as well as to examine whether they are enriched on small chromosomes. Previous studies have determined the position of centromeres for *T. cruzi* chromosomes by a functional mapping and measuring of topoisomerase II activity (a biochemical marker for active centromeres) (Obado et al., 2005, 2007) and *T. brucei* megabase chromosomes by topoisomerase II activity (Obado et al., 2007; Echeverry et al., 2012). The identified centromeric regions contain various degenerate retroelements in both organisms. Furthermore, the *T. brucei* megabase chromosomes also contain repetitive sequences whose units are relatively AT rich (except for those on chromosome 3). It remains to be shown whether kinetochore assembly in fact occurs at the mapped regions, and if so, exactly where kinetochores are assembled. Site-specific topoisomerase II activity was not identified for the intermediate or minichromosomes (Obado et al., 2007), and it remains unknown whether these chromosomes utilize the same segregation machinery (Gull et al., 1998).

To address these questions, we performed chromatin immunoprecipitation of YFP-tagged KKT proteins followed by deep-sequencing (ChIP-seq). We chose KKT2 and KKT3 because they have punctate signals throughout the cell cycle and therefore may directly bind DNA (see below). A histone H3 variant, H3v, was also analyzed for comparison. Sequencing reads were mapped to a reference genome that contains 11 megabase chromosomes, as well as a model minichromosome that mostly consists of the 177 bp repeats (see Experimental Procedures). The results were normalized based on the number of reads from each input sample, and we calculated enrichment ratios for nonoverlapping windows of 150 bp in size. Centromeres for chromosomes 9, 10, and 11 are not in the genome assembly, so we focused on chromosomes 1–8 and the model minichromosome.

We found that both YFP-KKT2 and YFP-KKT3 have a strong peak on each megabase chromosome that corresponds to the mapped centromeric region (Figures 2A and S2). YFP-H3v did not have specific enrichment at centromeric regions but was enriched at transcription termination sites as previously reported (Siegel et al., 2009) (Figure 2B). These results show that KKT2 and KKT3 are enriched at the identified centromeric regions in the megabase chromosomes and thus confirm that they are bona fide kinetochore proteins.

For seven out of eight megabase chromosomes (chromosomes 1, 2, and 4–8), the highly enriched regions correspond

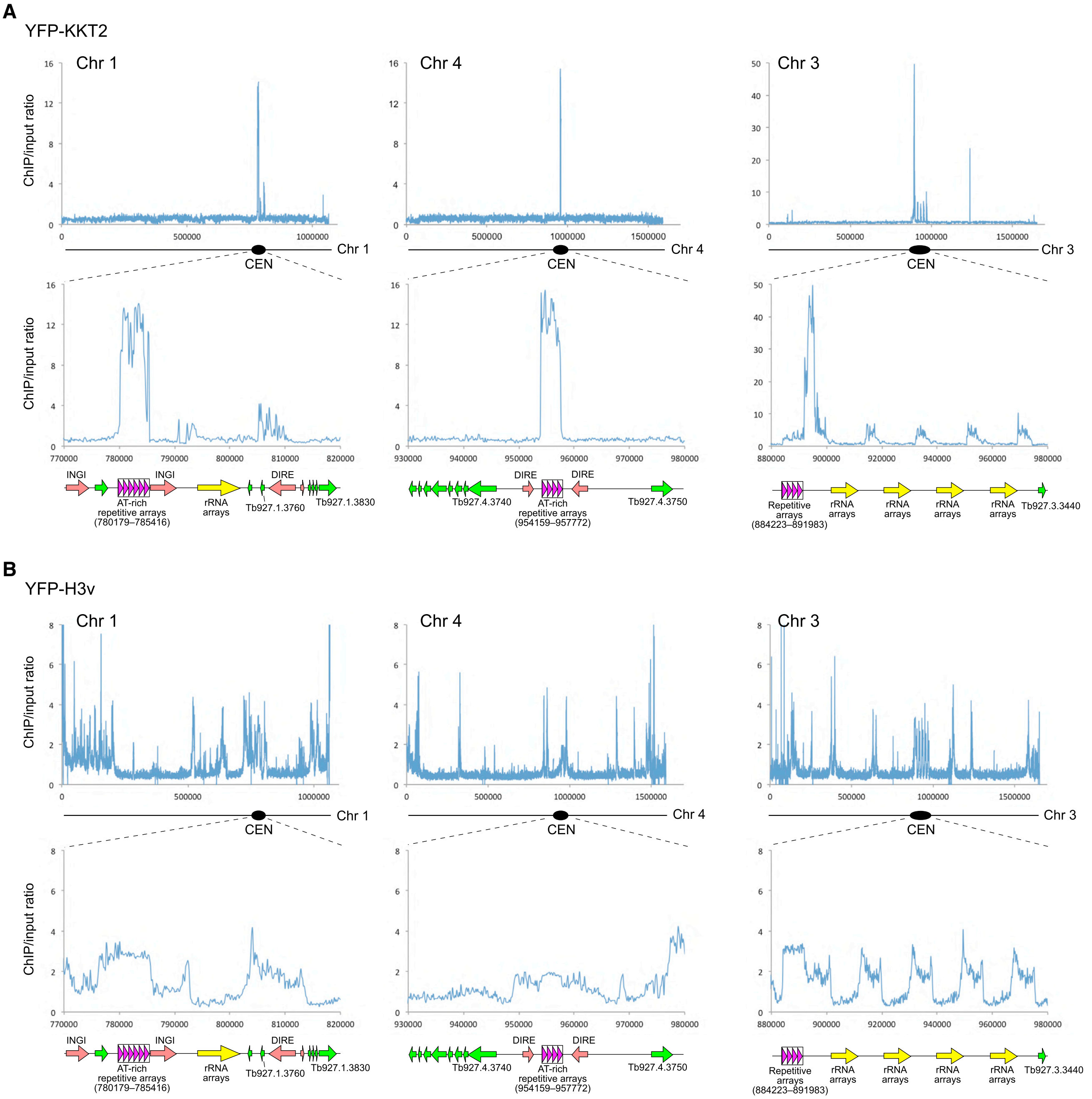

Figure 2. KKT2 Is Enriched at Megabase Chromosome Centromeres

(A) ChIP-seq data for YFP-KKT2. Top panels show chromosome-wide views of enrichment ratio, whereas bottom panels show a zoomed-in view of the centromeric region. Data for more megabase chromosomes and a model minichromosome, as well as our interpretation of several noncentromeric peaks, are shown in Figure S2.

(B) ChIP-seq data for the YFP-H3v control.

See also Figure S2 and Table S5.

to the AT-rich repetitive arrays (Figures 2A, S2A, and S2B), suggesting that kinetochores are assembled onto repetitive sequences, as in humans (Hayden et al., 2013). In contrast, chromosome 3 had a strong enrichment adjacent to the repetitive sequences (Figures 2A and S2B). It is interesting that neither the repetitive arrays nor the enriched regions are AT rich for this chromosome.

We observed some enrichment on the minichromosome 177 bp repeats for the two KKT proteins, as well as H3v (Figure S2C). The core of minichromosomes consists of the

177 bp repeats in a palindromic manner with an inversion point in the middle (Wickstead et al., 2004). Although the nature of highly homogenous sequences does not allow us to determine where along the minichromosomes KKT proteins are enriched, this result implies either that kinetochores are also assembled onto minichromosomes or that minichromosomes are in close proximity to the kinetochores assembled on megabase chromosomes, possibly hijacking them to facilitate the segregation of minichromosomes.

KKT Proteins Are Essential for Chromosome Segregation

We next examined the biological importance of KKT proteins by inducible RNAi-mediated knockdown. We focused on KKT2, KKT7, KKT9, KKT11, KKT12, and KKT10/KKT19 for which a reasonable level of depletion of protein was achieved at 48 hr postinduction (Figure 3A). Upon induction of RNAi, growth retardation was observed in each case, albeit at a varying degree (Figure 3B). As expected, we detected abnormal DNA content and morphology in RNAi-induced cells (Figures 3C and 3D). The strongest effect was observed on KKT9 RNAi cells, which is consistent with the greatest growth defect (Figure 3B). We also monitored the position of kinetochores at an earlier time point (24 hr) by performing RNAi in YFP-KKT2 cell lines and observed lagging kinetochores in anaphase cells (Figures 3E and 3F). Because megabase chromosomes account for ~80% of nuclear DNA, these results suggest that KKT proteins are essential for the faithful segregation of megabase chromosomes. To confirm this, we performed a fluorescence in situ hybridization (FISH) analysis using a CEN3 repeat probe to monitor the fate of chromosome 3 homologs and found that 15% of anaphase cells had missegregation in KKT10/KKT19 RNAi-induced cells (0% in control, n = 40 each) (Figure 3G).

Because minichromosomes also have enrichment of KKT proteins, we examined whether the segregation of minichromosomes is also affected. Minichromosomes were monitored by FISH using a 177 bp probe (Ersfeld and Gull, 1997). We found that 93% of anaphase cells had abnormal signals in RNAi-induced cells, compared to 10% in control cells (n = 30 each) (Figure 3H). These results reveal that KKT proteins are essential for the segregation of both megabase chromosomes and minichromosomes.

Predictions of Function

Having established that KKT proteins are kinetochore proteins that play crucial roles in chromosome segregation, we next aimed to gain insights into the potential functions of individual KKT proteins from their localization patterns and bioinformatic analyses. Studies of conventional kinetochore proteins have established that their functions are often manifested in localization patterns. For example, in humans, the CENP-A protein that directly binds DNA is constitutively localized at centromeres, whereas the microtubule-binding Ndc80 subcomplex localizes at kinetochores from the onset of mitosis until the end of anaphase (Cheeseman and Desai, 2008). We observed the following patterns for the KKT proteins (Figures 4 and S3): constitutive (therefore potential DNA-binding candidates): KKT2, KKT3, and KKT4; S phase specific: KKT13; detectable from S phase until the end of anaphase (structural role and/or microtubule-binding candidates): KKT1, KKT5, KKT6, KKT7, KKT16, KKT17, and KKT18 (it is noteworthy that KKT16, KKT17, and KKT18 additionally have diffuse nuclear signals in G1); from G2/M until the end of anaphase (microtubule-binding candidates): KKT14 and KKT15; from S phase until the anaphase onset (regulator of kinetochore function candidates): KKT8, KKT9, KKT10, KKT11, KKT12, and KKT19. We speculate that those proteins that localize until the end of anaphase likely constitute the core kinetochore (KKT1, 2, 3, 4, 5, 6, 7, 14, 15, 16, 17, and 18).

Studies from other eukaryotes also established that kinetochores often consist of stable subcomplexes that form functional units and have similar localization patterns (Cheeseman and Desai, 2008). From our affinity purification/MS results, we deduced the following subcomplexes for the *T. brucei* kinetochore (Table S3): KKT14-KKT15 subcomplex, KKT16-KKT17-KKT18 subcomplex, and KKT6-KKT7-KKT8-KKT9-KKT10-KKT11-KKT12-KKT19 subcomplex. The fact that these proteins have largely similar localization patterns supports the assignment of these putative subcomplexes (Figure 4).

Sequence analysis of the KKT proteins revealed that the following domains and motifs are conserved among kinetoplastids (Figure 5A): the BRCA1 C terminus (BRCT) domain (KKT4), the forkhead-associated (FHA) domain (KKT13), the WD40-like domain (KKT15), protein kinase domains (KKT2, KKT3, KKT10, and KKT19), cysteine-rich domains (KKT2, KKT3), and a putative PP1-binding motif (KKT7). The BRCT and FHA domains typically function as phospho-Ser/Thr and phospho-Thr binding domains, respectively, and are found in many DNA damage response proteins (Reinhardt and Yaffe, 2013). It is noteworthy that BRCT and FHA domains are not found in any known conventional kinetochore proteins. The WD40 domain is one of the most abundant domains in eukaryotic genomes and is found in proteins involved in a large variety of cellular processes (Reinhardt and Yaffe, 2013).

KKT10 and KKT19 exhibit a high degree of similarity at the protein level, as do KKT2 and KKT3 and KKT17 and KKT18, suggesting that these pairs likely arose from gene duplication events. Although KKT10 and KKT19 have previously been classified as members of the CLK/Lammer subfamily in the CMGC family (Parsons et al., 2005), there are significant differences between KKT10/KKT19 and the human or *Arabidopsis* CLK/Lammer kinases (Figure S4), implying that KKT10/KKT19 may have adapted to carry out kinetochore functions in kinetoplastids. Interestingly these kinases (named as TbCLK1 and TbCLK2 in that study) were recently identified as targets of a fungal natural product Hypothemycin (Nishino et al., 2013), demonstrating the potential of trypanosome kinetochore kinases as drug targets.

KKT2 and KKT3 possess residues characteristic of active eukaryotic protein kinases but do not have a clear affiliation to any known group or family (Parsons et al., 2005), suggesting that these proteins are likely to be kinetoplastid specific. Interestingly, these proteins also possess cysteine-rich domains (Figure 5A), in which classic zinc-finger motifs can be recognized (Figure 5B). Furthermore, several DNA-binding motifs (SPKK [Suzuki, 1989] and AT-hook [Aravind and Landsman, 1998]) are found in KKT2 and KKT3 in some kinetoplastids, although not strictly conserved across kinetoplastids (Figure 5C).

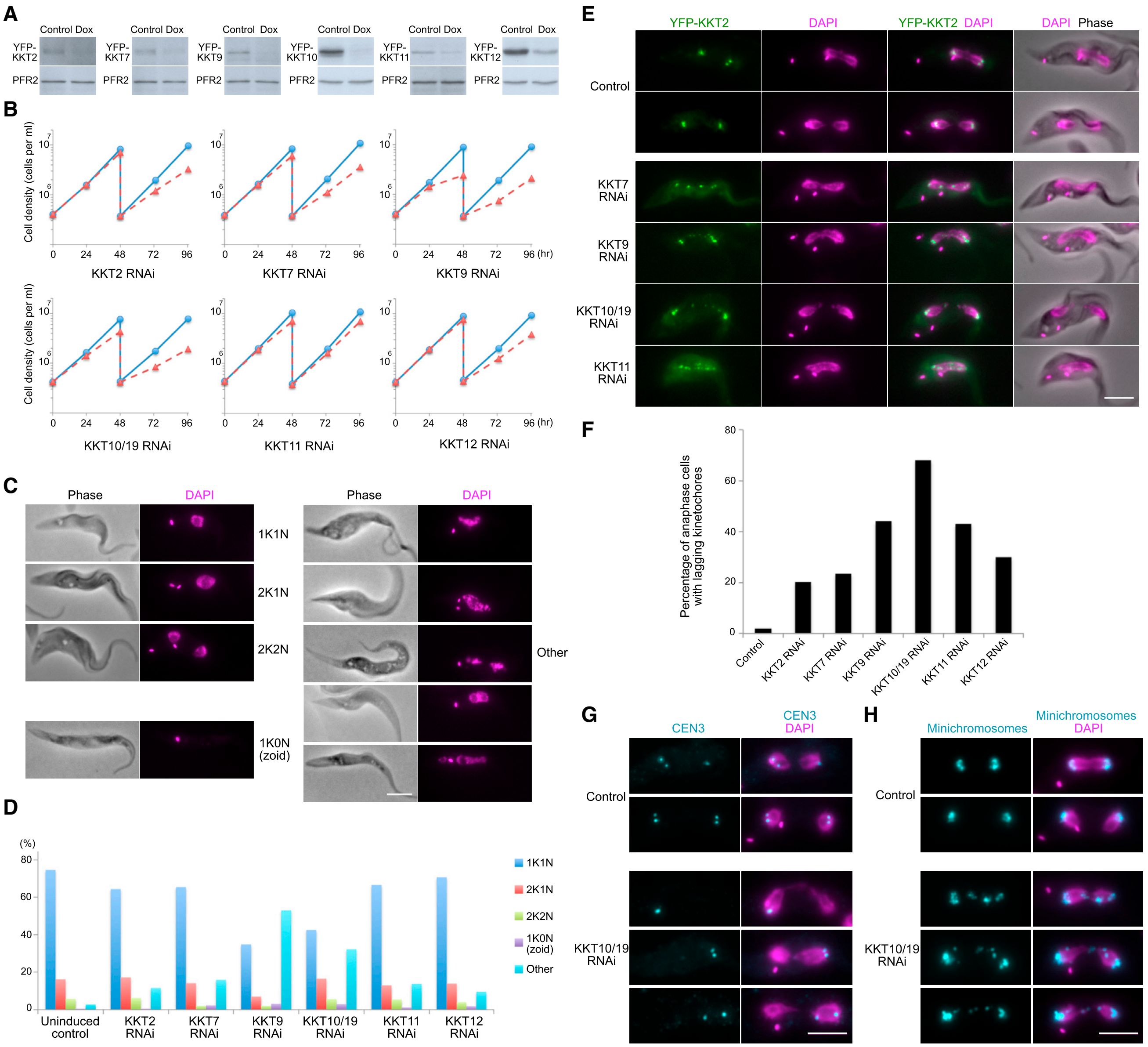

Figure 3. KKTs Are Essential for Faithful Chromosome Segregation

(A) Immunoblots against YFP-tagged KKT proteins show reduction upon RNAi induction with doxycycline. The PFR2 protein was used as a loading control.

(B) RNAi-mediated knockdown of KKT proteins affects cell growth. Blue lines indicate noninduced controls, and dotted red lines indicate RNAi-induced cells. Note that the KKT10 RNAi construct also targets KKT19.

(C) Examples of normal and abnormal cells stained with DAPI. Cells in "other" category show an abnormal number and/or shape of nuclear DNA. Zoid (1K0N) cells lack a nuclear DNA. Phase contrast images are shown in the left panels.

(D) Quantification of cells with indicated DNA contents (n = 500 each). Data for (A)–(D) were collected from cells at 48 hr postinduction.

(E) Examples of anaphase cells that express YFP-KKT2 with RNAi of indicated KKT proteins.

(F) Quantification of anaphase cells with lagging kinetochores (n = 100 each).

(G) Fluorescence in situ hybridization analysis of the chromosome 3 homologous pair.

(H) Fluorescence in situ hybridization analysis of all minichromosomes. Data for (E)–(H) were collected from cells at 24 hr postinduction. Bars, 5 μm.

Together with their constitutive localization pattern (Figure 4), we speculate that KKT2 and KKT3 are loaded onto centromeric DNA via the cysteine-rich domains, SPKK and/or AT-hook, to modify other proteins via the unique kinase domains, contributing to the establishment of kinetochores.

Taken together, our bioinformatic analysis failed to find any evidence that KKT proteins are similar to conventional kinetochore proteins at the primary sequence level. Although this by itself does not mean that kinetoplastid kinetochores are completely different, it is striking that all of the features we detect imply

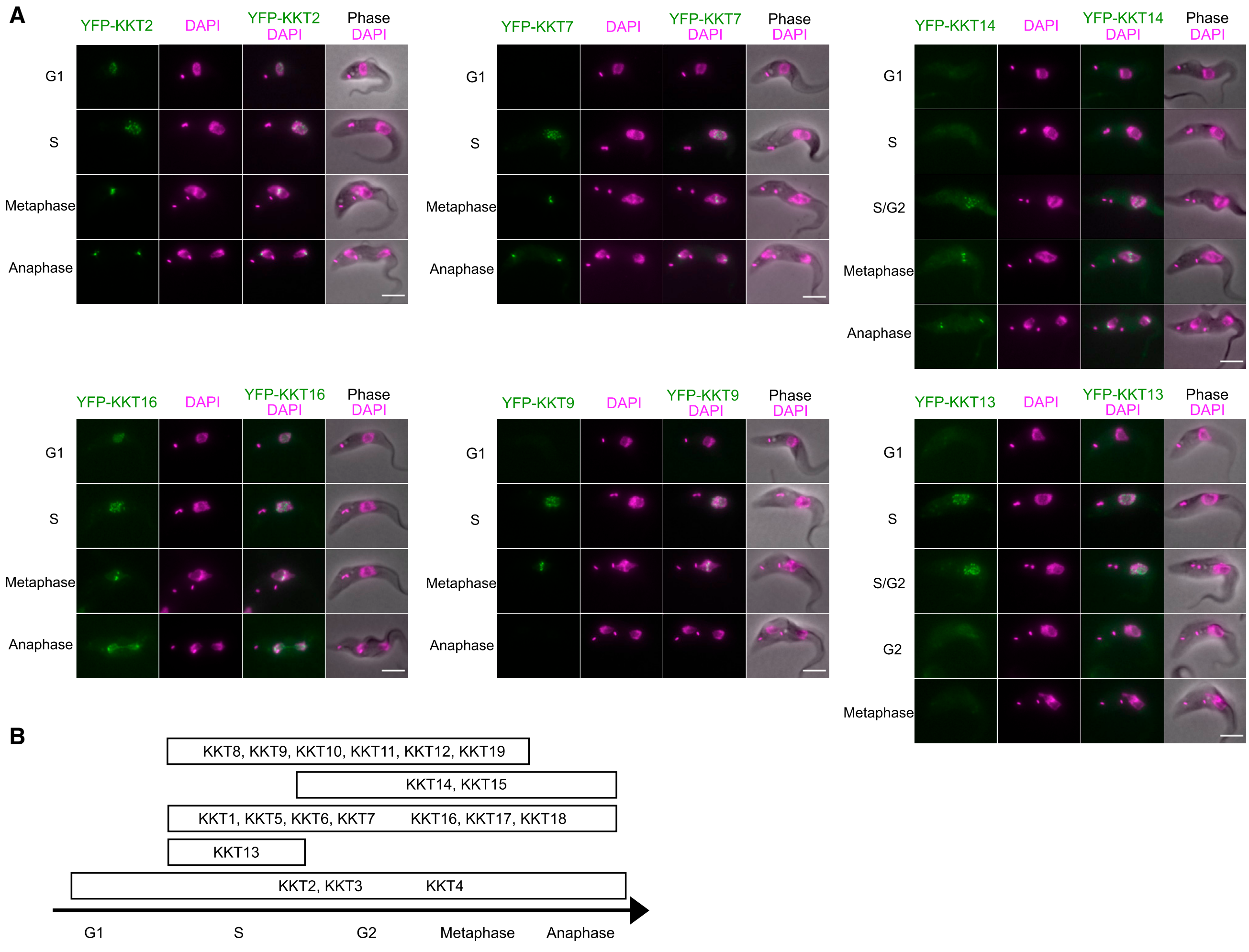

Figure 4. KKT Proteins Show Differential Localization Timings

(A) Examples of cells expressing YFP-tagged KKT proteins. Bars, 5 μm. Data for other KKT proteins are shown in Figure S3.

(B) Summary of localization patterns.

See also Figure S3 and Table S3.

difference, not similarity. The simplest interpretation is that kinetoplastids contain unconventional kinetochores composed of distinct kinetochore proteins.

DISCUSSION

Accurate transmission of genetic material in eukaryotes depends on the attachment of dynamic spindle microtubules to chromosomes via the macromolecular kinetochore complexes. Available evidence suggests that spindle microtubules composed of α/β tubulins are ubiquitously used in all eukaryotes studied thus far (Wickstead and Gull, 2011; Drechsler and McAinsh, 2012). In contrast, it was previously not clear whether all eukaryotes utilize similar kinetochore proteins because none of the conventional kinetochore components were identifiable in any kinetoplastid genome. Our identification of 19 kinetochore proteins in *T. brucei* has revealed that kinetoplastid kinetochores are composed of proteins that are distinct from conventional kinetochore proteins in other eukaryotes. This new group of proteins may therefore constitute an attractive drug target for kinetoplastid diseases, such as sleeping sickness and nagana caused by *T. brucei*, Chagas disease caused by *T. cruzi*, and leishmaniasis caused by *Leishmania* species (Stuart et al., 2008). Further studies will be required to explore the unconventional kinetochores as a means to combat these diseases.

The goal of the eukaryotic kinetochore is to mediate the interaction between DNA and microtubules. Therefore, understanding how KKT proteins achieve these tasks in kinetoplastids will contribute to a better understanding of how conventional kinetochores function. For example, we still do not know why CENP-A is so widely used in eukaryotes, despite the fact that CENP-A is not strictly essential for building functional kinetochores (Hori et al., 2013). The current prevailing idea is that CENP-A forms a centromere-specific chromatin environment that somehow acts as an epigenetic marker for kinetochore assembly (Nechemia-Arbely et al., 2012; Müller and Almouzni,

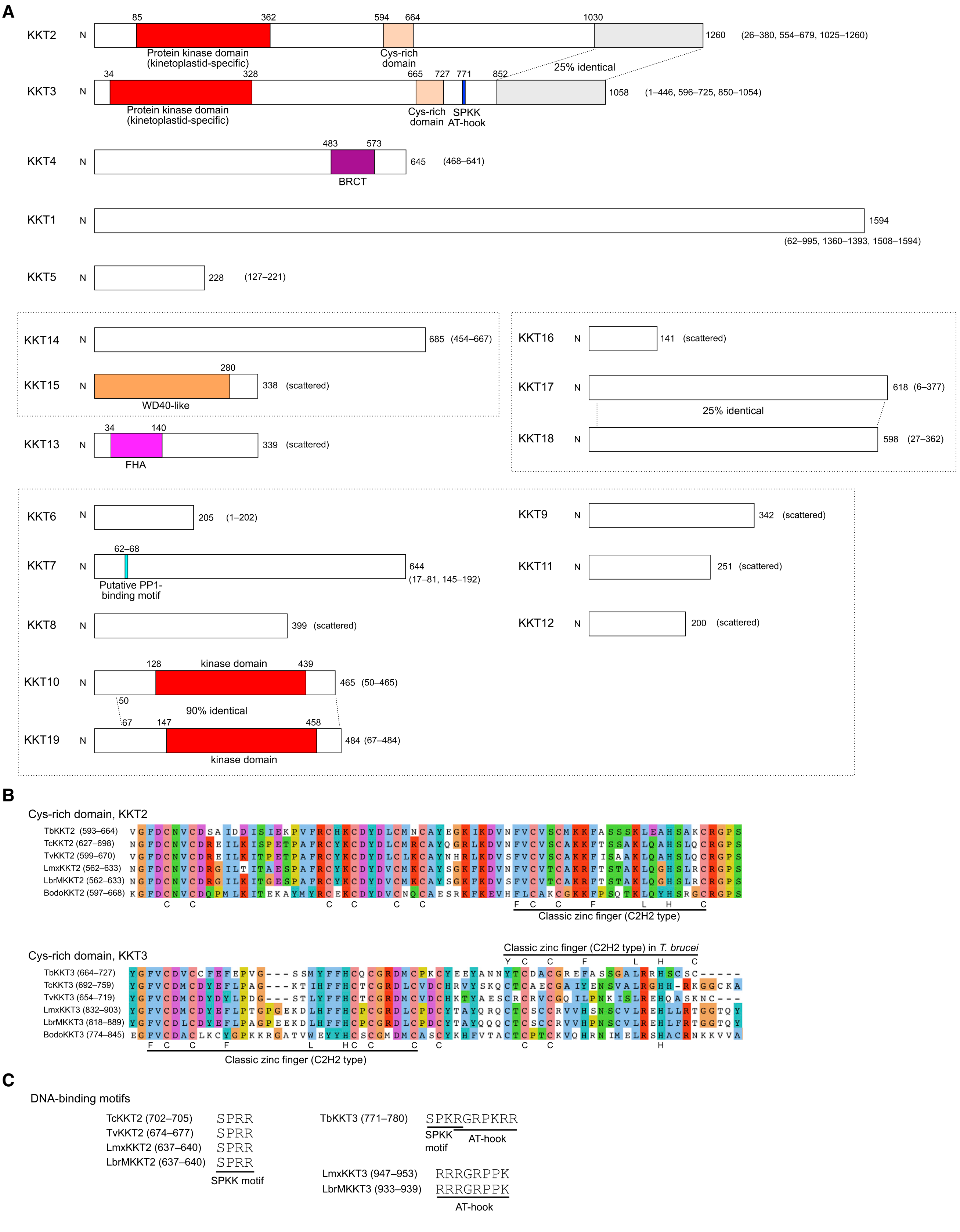

(legend on next page)

2013). Because kinetoplastids do not possess CENP-A by nature, it is not clear whether their centromere identity is epigenetically defined by a distinct mechanism or how the kinetochore assembly site is determined. By understanding the CENP-A-independent kinetochores in kinetoplastids, we may obtain insights into the specialty of CENP-A.

There is an intimate relationship between repetitive sequences, the endogenous RNAi machinery, and the kinetochore assembly in many species (Buscaino et al., 2010). The endogenous RNAi system is important for faithful chromosome segregation in *T. brucei* (Durand-Dubief et al., 2007), and noncoding RNAs from some, but not all, centromeric repeats have been detected (Tschudi et al., 2012). It will be important to determine whether (and how) the RNAi system contributes to the deposition of kinetochore proteins at centromeres in *T. brucei*. In contrast, *T. cruzi* does not possess an endogenous RNAi system, and its centromeres are devoid of repetitive sequences (Obado et al., 2005). It will also be interesting to reveal how *T. cruzi* determines the kinetochore assembly sites in the absence of an RNAi system.

T. brucei does not appear to possess a functional spindle checkpoint system that monitors the kinetochore-microtubule attachment and regulates the activation of the anaphase promoting complex (Ploubidou et al., 1999; Akiyoshi and Gull, 2013). However, we found that KKT4 copurifies with several APC/C subunits (Table S2), raising a possibility that this kinetochore protein may directly communicate with the APC/C. It is interesting that KKT4 signal is not mitosis specific but is found throughout the cell cycle (and thus may locate close to DNA rather than microtubules). Gaining insights into the functions of KKT4 may lead to a better understanding of the APC/C regulatory mechanism, as well as the nature of signals transmitted from kinetochores to regulate the APC/C.

Determining the position of the root of the eukaryotic tree of life remains an unresolved problem (Embley and Martin, 2006; Walker et al., 2011). Among several competing hypotheses (e.g., Stechmann and Cavalier-Smith, 2002; Rogozin et al., 2009; Katz et al., 2012), it has been proposed, based on unique mitochondrial cytochromes c/c1 and the absence of a recognizable biogenesis apparatus for these proteins (Allen et al., 2008), that Euglenozoa (a phylum that includes kinetoplastids) may represent extremely early or the earliest-branching eukaryotes (Cavalier-Smith, 2010). Therefore, it is possible that kinetoplastids evolved the KKT-based kinetochore system early in the eukaryotic history, whereas other eukaryotes evolved a system utilizing conventional kinetochore proteins. A corollary is that this controversial hypothesis that roots the base of the eukaryotic tree between Euglenozoa (or deep within the Euglenozoa tree) and all other eukaryotes now receives support from two very distinct properties (mitochondrial cytochromes and kinetochores), as well as many others (Cavalier-Smith, 2010, 2013). More work on kinetoplastids and other Euglenozoa species is very much warranted to further test the validity of this hypothesis. However, even if this rooting is correct, we would not be able to tell what kind of kinetochores the last eukaryotic common ancestor (LECA) possessed. It might be that the LECA possessed a conventional kinetochore system, which was later replaced by the KKT-based system in kinetoplastids (Figure S5A). Or it might be that the LECA utilized the KKT system, but only kinetoplastids retained it, whereas other eukaryotes lost it and developed a conventional kinetochore system (Figure S5B). Alternatively, the LECA might have possessed a hitherto-unknown type of kinetochores (Figure S5C). Whatever the evolutionary history might be, understanding the KKT-based kinetochores in kinetoplastids should lead to a better understanding of the chromosome segregation machinery in eukaryotes.

EXPERIMENTAL PROCEDURES

Cells

All cell lines used in this study were derived from *T. brucei* SmOxP927 procyclic form cells (TREU 927/4 expressing T7 RNA polymerase and the tetracycline repressor to allow inducible expression) (Poon et al., 2012) and are listed in Table S4. Plasmids and primers used in this study are also listed in Table S4. Cells were grown at 28°C in SDM-79 medium supplemented with 10% (v/v) heat-inactivated fetal calf serum (Brun and Schönenberger, 1979). Cell growth was monitored using a CASY cell counter and analyzer system (Roche). For induction of RNAi, doxycycline was added to the medium to a final concentration of 1 μg/ml. Endogenous YFP and tdTomato tagging was performed using the pEnT5-Y vector (Kelly et al., 2007) and pBA148, respectively. For generation of inducible RNAi cell lines, ~500 bp fragments were amplified from genomic DNA and cloned into the p2T7-177 vector (Wickstead et al., 2002). Details on plasmid construction are described in the Extended Experimental Procedures. Plasmids linearized by NotI site were transfected to trypanosomes by electroporation into an endogenous locus (pEnT5-Y and pBA148 derivatives) or 177 bp repeats on minichromosomes (p2T7-177 derivatives). Transfected cells were selected by the addition of 25 μg/ml hygromycin (pEnT5-Y derivatives), 10 μg/ml blasticidin (pBA148 derivatives), or 5 μg/ml phleomycin (p2T7-177 derivatives).

Fluorescence Microscopy

For the analysis of fluorescently tagged proteins or DNA contents, cells were washed once with PBS, settled onto glass slides, and fixed with 4% paraformaldehyde in PBS for 5 min. Cells were then permeabilized with 0.1% NP-40 in PBS for 5 min and embedded in mounting media (1% 1,4-Diazabicyclo [2.2.2]octane (DABCO), 90% glycerol, and 50 mM sodium phosphate [pH 8.0]) containing 100 ng/ml DAPI. FISH was carried out as described (Ersfeld and Gull, 1997) using digoxigenin-labeled probes against 177 bp repeats (Ersfeld and Gull, 1997) (for minichromosomes) or 120 bp CEN3 repetitive arrays (Obado et al., 2007) (for the chromosome 3 homologous pair). Images were captured on a fluorescence microscope (Leica Microsystems) equipped with an Orca cooled charge-coupled device (CCD) camera (Hamamatsu Photonics), and processed in ImageJ (Schneider et al., 2012).

Immunoprecipitation

We developed an immunoprecipitation method for trypanosome kinetochore proteins based on our kinetochore purification protocol in budding yeast

Figure 5. Domain Organization of *T. brucei* KKT Proteins

(A) Schematic representation of *T. brucei* KKT proteins. Identified domains and motifs, as well as blocks that are highly conserved among kinetoplastids (given in parenthesis), are shown. Putative subcomplexes are grouped in dotted boxes.

(B) Alignment of cysteine-rich domains of KKT2 and KKT3 from six kinetoplastid species (*T. brucei*, *T. cruzi*, *T. vivax*, *L. mexicana*, *L braziliensis*, and *B. saltans*).

(C) DNA-binding motifs found in KKT2 and KKT3 proteins.

See also Figures S4 and S5 and Table S4.

(Akiyoshi et al., 2010). Typically, 400 ml cultures of asynchronously growing cells were harvested at 1.2×10^7 cells/ml. Cells were pelleted by centrifugation (900 g, 10 min), washed once with PBS, and extracted in PEME (100 mM PIPES-NaOH [pH 6.9], 2 mM EGTA, 1 mM $MgSO_4$, and 0.1 mM EDTA) with 1% NP-40 and protease inhibitors (Leupeptin, Pepstatin, E-64, 10 μg/ml each, and 0.2 mM PMSF) and phosphatase inhibitors (1 mM sodium pyrophosphate, 2 mM Na-beta-glycerophosphate, 0.1 mM Na_3VO_4, 5 mM NaF, and 100 nM microcystin-LR) for 5 min at room temperature, followed by centrifugation (1,800 g, 15 min). Samples were kept on ice from now on. The pelleted fractions that contain kinetochore proteins were resuspended in modified buffer H (BH)/0.15 (25 mM HEPES [pH 8.0], 2 mM $MgCl_2$, 0.1 mM EDTA [pH 8.0], 0.5 mM EGTA [pH 8.0], 1% NP-40, 150 mM KCl, and 15% glycerol) containing protease inhibitors and phosphatase inhibitors. Samples were sonicated to solubilize kinetochore proteins (12 s, 3 times with 1 min interval on ice), producing "input" extract samples. 12 μg of mouse monoclonal anti-GFP antibodies (Roche, 11814460001) that had been preconjugated with 60 μl slurry of Protein-G magnetic beads (Dynal) with dimethyl pimelimidate (as described in Unnikrishnan et al. [2012]) were incubated with input extracts for 3 hr with constant rotation, followed by four washes with modified BH/0.15 containing protease inhibitors, phosphatase inhibitors, and 2 mM dithiothreitol (DTT). Beads were further washed three times with pre-elution buffer (50 mM Tris-HCl [pH 8.3], 75 mM KCl, and 1 mM EGTA). Associated proteins were gently eluted from the beads by agitation in 60 μl of elution buffer (0.1% Rapigest and 50 mM Tris-HCl [pH 8.3]) for 25 min at room temperature. 10 μl of samples were analyzed by immunoblots (using monoclonal anti-GFP or anti-Ty antibodies) and Sypro-Ruby staining. The rest of samples (50 μl) were used to identify copurifying proteins by MS as described below. SDS-PAGE and immunoblots were performed by standard methods using the following mouse monoclonal antibodies: anti-GFP (Roche, 11814460001) or BB2 (anti-Ty) (Bastin et al., 1996) for TY-YFP-tagged KKT proteins and L8C4 (anti-PFR2) (Kohl et al., 1999) for a loading control.

Mass Spectrometry

The samples were incubated at 100°C for 5 min. Proteins were reduced with 5 mM DTT at 37°C for 30 min and alkylated with 10 mM iodoacetamide at 37°C for 30 min. The reaction was quenched by adding 10 mM DTT at 37°C for 30 min, and 100 μl of 20 mM Tris-HCl (pH 8.3) was added. Proteins were digested overnight at 37°C with 0.4 μg of trypsin (Promega). Then formic acid was added to 2%, and the samples were incubated at 37°C for 30 min to cleave the detergent Rapigest, followed by centrifugation for 10 min. The supernatant was desalted over a C18 column and analyzed by electrospray tandem mass spectrometry over a 40 min gradient using an LTQ XL-Orbitrap (Thermo Scientific) at the Central Proteomics Facility (http://www.proteomics.ox.ac.uk, Sir William Dunn School of Pathology, University of Oxford).

Data analysis was performed by using the central proteomics facilities pipeline (CPFP) (Trudgian et al., 2010). Peptides were identified by searching MS/MS spectra against the *T. brucei* protein database with Mascot (Matrix Science), OMSSA (Geer et al., 2004), and X!Tandem (Craig and Beavis, 2004) with carbamidomethyl cysteine as fixed modification. Up to two missed cleavages were allowed. Oxidized-methionine and phosphorylation were searched as variable modifications. Mass tolerances for MS and MS/MS peak identifications were 20 ppm and 0.5 Da, respectively. Proteins identified with at least two peptides were considered and shown in Table S2. Raw MS data are available upon request.

Bioinformatics

Sources of predicted protein database used for the search of conventional kinetochore proteins in various organisms from the six eukaryotic supergroups (Walker et al., 2011) are listed in Table S4. Wherever possible, protein names searchable in the NCBI database are listed in Table S1. Putative CENP-A homologs were identified as reported previously (Talbert et al., 2009). Putative homologs for conventional kinetochore proteins (CENP-C, Ndc80, Nuf2, Spc24 and Spc25) were identified using HMMER (version 3.0) (Eddy, 1998; Finn et al., 2011). Pairwise sequence alignment and motif search were performed by EMBL-EBI tools (McWilliam et al., 2013) and Pfam (Punta et al., 2012), as well as manual inspection. Multiple sequence alignment was performed with MAFFT (version 7) (Katoh and Standley, 2013) and visualized with Clustalx coloring scheme in Jalview (version 2.8) (Waterhouse et al., 2009). Genome sequences for nonkinetoplastid Euglenozoa are currently not available, and we therefore do not know whether KKT-based kinetochores are conserved across Euglenozoa.

ChIP-Seq

ChIP was carried out essentially as described (Siegel et al., 2009) using 10 times more cells (1×10^9 cells for each experiment). Briefly, cells expressing either YFP-KKT2, YFP-KKT3, or YFP-H3v were fixed with 1% formaldehyde for 20 min at room temperature and sonicated to prepare input chromatin fragments, and YFP-tagged proteins were immunoprecipitated with rabbit polyclonal anti-GFP antibodies (Invitrogen, A11122) that were preconjugated with Protein-A magnetic beads (Dynal), followed by DNA purification. Single-end sequencing (49 bp sequence tag) was carried out on a HiSeq2000 Illumina platform at BGI Hong Kong. Both input DNA and ChIP DNA were sequenced in each case. Reads with adaptors, reads with unknown nucleotides larger than 5%, and reads with low quality (more than 20% of the bases' qualities are less than 10 in a read) were removed to provide clean reads (see Table S5 for statistics). Sequence tags were mapped using Burrows-Wheeler Aligner (version 0.7.4) (Li and Durbin, 2009), allowing up to two mismatches to the *T. brucei* 927 genome (version 5.0: note that centromeres of chromosomes 9, 10, and 11 are not in the genome assembly in this version) supplemented with a contig tryp_X-284f09.p1c (42,529 bp) that consists mostly of the 177 bp repeat sequences (and thus is very likely to be derived from a minichromosome) to examine the enrichment ratio on 177 bp sequences. When reads map to multiple locations in the reference genome (due to the presence of identical sequences), the program randomly chooses the hits. The following centromere repeat unit pairs have a similarity level higher than 96% (Obado et al., 2007), and we therefore cannot distinguish them in our analysis: between chromosome 10 and chromosomes 4/9/11 and between chromosome 4 and chromosome 9. Other pairs are less than 93% identical. The SAMtools program (Li et al., 2009) was used to generate bam files, and tag counting was done by BEDTools (Quinlan and Hall, 2010) (coverageBed) using 150 bp nonoverlapping windows. The ChIP/input ratio was then calculated for each window, and the results were visualized in Excel. To reduce noise, we ignored a few windows with less than 10 reads in the input. Background levels for each protein were calculated for 550,000–750,000 of chromosome 4 and are shown in Figure S2C (KKT2: 0.59; KKT3: 0.40; H3v: 0.41).

ACCESSION NUMBERS

NGS sequence data are deposited in the SRA database under accession number SRP031518.

SUPPLEMENTAL INFORMATION

Supplemental Information includes Extended Experimental Procedures, five figures, and five tables and can be found with this article online at http://dx.doi.org/10.1016/j.cell.2014.01.049.

ACKNOWLEDGMENTS

We thank Kim Nasmyth, Stuart Ferguson, Sue Biggins, and Hannah Long for comments on the manuscript. We also thank Vladimir Varga for discussion; Jonathan Higgins for advice on kinases; Takashi Sutani, Nicolai Siegel, and Mike Fiebig for advice on ChIP-seq; and the Central Proteomics Facility, Benjamin Thomas, and Gabriela Ridlova for mass spectrometry analysis. B.A. was supported by postdoctoral fellowships from the EMBO and Human Frontier Science Program and is currently supported by a Sir Henry Dale Fellowship jointly funded by the Wellcome Trust and the Royal Society (grant 098403/Z/12/Z), as well as a Wellcome-Beit Prize Fellowship (grant 098403/Z/12/A). Research in the Gull laboratory is supported by the Wellcome Trust. The authors declare no competing financial interests.

Received: December 3, 2013
Revised: January 6, 2014
Accepted: January 10, 2014
Published: February 27, 2014

REFERENCES

Akiyoshi, B., and Gull, K. (2013). Evolutionary cell biology of chromosome segregation: insights from trypanosomes. Open Biol. *3*, 130023.

Akiyoshi, B., Sarangapani, K.K., Powers, A.F., Nelson, C.R., Reichow, S.L., Arellano-Santoyo, H., Gonen, T., Ranish, J.A., Asbury, C.L., and Biggins, S. (2010). Tension directly stabilizes reconstituted kinetochore-microtubule attachments. Nature *468*, 576–579.

Allen, J.W.A., Ferguson, S.J., and Ginger, M.L. (2008). Distinctive biochemistry in the trypanosome mitochondrial intermembrane space suggests a model for stepwise evolution of the MIA pathway for import of cysteine-rich proteins. FEBS Lett. *582*, 2817–2825.

Altschul, S.F., Madden, T.L., Schäffer, A.A., Zhang, J., Zhang, Z., Miller, W., and Lipman, D.J. (1997). Gapped BLAST and PSI-BLAST: a new generation of protein database search programs. Nucleic Acids Res. *25*, 3389–3402.

Aravind, L., and Landsman, D. (1998). AT-hook motifs identified in a wide variety of DNA-binding proteins. Nucleic Acids Res. *26*, 4413–4421.

Archer, S.K., Inchaustegui, D., Queiroz, R., and Clayton, C. (2011). The cell cycle regulated transcriptome of *Trypanosoma brucei*. PLoS ONE *6*, e18425.

Bastin, P., Bagherzadeh, Z., Matthews, K.R., and Gull, K. (1996). A novel epitope tag system to study protein targeting and organelle biogenesis in *Trypanosoma brucei*. Mol. Biochem. Parasitol. *77*, 235–239.

Berriman, M., Ghedin, E., Hertz-Fowler, C., Blandin, G., Renauld, H., Bartholomeu, D.C., Lennard, N.J., Caler, E., Hamlin, N.E., Haas, B., et al. (2005). The genome of the African trypanosome *Trypanosoma brucei*. Science *309*, 416–422.

Biggins, S. (2013). The composition, functions, and regulation of the budding yeast kinetochore. Genetics *194*, 817–846.

Brun, R., and Schönenberger. (1979). Cultivation and in vitro cloning or procyclic culture forms of *Trypanosoma brucei* in a semi-defined medium. Short communication. Acta Trop. *36*, 289–292.

Buscaino, A., Allshire, R., and Pidoux, A. (2010). Building centromeres: home sweet home or a nomadic existence? Curr. Opin. Genet. Dev. *20*, 118–126.

Cavalier-Smith, T. (2010). Kingdoms Protozoa and Chromista and the eozoan root of the eukaryotic tree. Biol. Lett. *6*, 342–345.

Cavalier-Smith, T. (2013). Early evolution of eukaryote feeding modes, cell structural diversity, and classification of the protozoan phyla Loukozoa, Sulcozoa, and Choanozoa. Eur. J. Protistol. *49*, 115–178.

Cheeseman, I.M., and Desai, A. (2008). Molecular architecture of the kinetochore-microtubule interface. Nat. Rev. Mol. Cell Biol. *9*, 33–46.

Craig, R., and Beavis, R.C. (2004). TANDEM: matching proteins with tandem mass spectra. Bioinformatics *20*, 1466–1467.

Daniels, J.-P., Gull, K., and Wickstead, B. (2010). Cell biology of the trypanosome genome. Microbiol. Mol. Biol. Rev. *74*, 552–569.

Drechsler, H., and McAinsh, A.D. (2012). Exotic mitotic mechanisms. Open Biol. *2*, 120140.

Durand-Dubief, M., Absalon, S., Menzer, L., Ngwabyt, S., Ersfeld, K., and Bastin, P. (2007). The Argonaute protein TbAGO1 contributes to large and mini-chromosome segregation and is required for control of RIME retroposons and *RHS* pseudogene-associated transcripts. Mol. Biochem. Parasitol. *156*, 144–153.

Echeverry, M.C., Bot, C., Obado, S.O., Taylor, M.C., and Kelly, J.M. (2012). Centromere-associated repeat arrays on *Trypanosoma brucei* chromosomes are much more extensive than predicted. BMC Genomics *13*, 29.

Eddy, S.R. (1998). Profile hidden Markov models. Bioinformatics *14*, 755–763.

Embley, T.M., and Martin, W. (2006). Eukaryotic evolution, changes and challenges. Nature *440*, 623–630.

Ersfeld, K. (2011). Nuclear architecture, genome and chromatin organisation in *Trypanosoma brucei*. Res. Microbiol. *162*, 626–636.

Ersfeld, K., and Gull, K. (1997). Partitioning of large and minichromosomes in *Trypanosoma brucei*. Science *276*, 611–614.

Finn, R.D., Clements, J., and Eddy, S.R. (2011). HMMER web server: interactive sequence similarity searching. Nucleic Acids Res. *39* (Web Server issue), W29–W37.

Geer, L.Y., Markey, S.P., Kowalak, J.A., Wagner, L., Xu, M., Maynard, D.M., Yang, X., Shi, W., and Bryant, S.H. (2004). Open mass spectrometry search algorithm. J. Proteome Res. *3*, 958–964.

Gull, K., Alsford, S., and Ersfeld, K. (1998). Segregation of minichromosomes in trypanosomes: implications for mitotic mechanisms. Trends Microbiol. *6*, 319–323.

Hayden, K.E., Strome, E.D., Merrett, S.L., Lee, H.-R., Rudd, M.K., and Willard, H.F. (2013). Sequences associated with centromere competency in the human genome. Mol. Cell. Biol. *33*, 763–772.

Hori, T., and Fukagawa, T. (2012). Establishment of the vertebrate kinetochores. Chromosome Res. *20*, 547–561.

Hori, T., Shang, W.-H., Takeuchi, K., and Fukagawa, T. (2013). The CCAN recruits CENP-A to the centromere and forms the structural core for kinetochore assembly. J. Cell Biol. *200*, 45–60.

Katoh, K., and Standley, D.M. (2013). MAFFT multiple sequence alignment software version 7: improvements in performance and usability. Mol. Biol. Evol. *30*, 772–780.

Katz, L.A., Grant, J.R., Parfrey, L.W., and Burleigh, J.G. (2012). Turning the crown upside down: gene tree parsimony roots the eukaryotic tree of life. Syst. Biol. *61*, 653–660.

Kelly, S., Reed, J., Kramer, S., Ellis, L., Webb, H., Sunter, J., Salje, J., Marinsek, N., Gull, K., Wickstead, B., and Carrington, M. (2007). Functional genomics in *Trypanosoma brucei*: a collection of vectors for the expression of tagged proteins from endogenous and ectopic gene loci. Mol. Biochem. Parasitol. *154*, 103–109.

Kohl, L., Sherwin, T., and Gull, K. (1999). Assembly of the paraflagellar rod and the flagellum attachment zone complex during the *Trypanosoma brucei* cell cycle. J. Eukaryot. Microbiol. *46*, 105–109.

Li, H., and Durbin, R. (2009). Fast and accurate short read alignment with Burrows-Wheeler transform. Bioinformatics *25*, 1754–1760.

Li, H., Handsaker, B., Wysoker, A., Fennell, T., Ruan, J., Homer, N., Marth, G., Abecasis, G., and Durbin, R.; 1000 Genome Project Data Processing Subgroup (2009). The Sequence Alignment/Map format and SAMtools. Bioinformatics *25*, 2078–2079.

Lowell, J.E., and Cross, G.A.M. (2004). A variant histone H3 is enriched at telomeres in *Trypanosoma brucei*. J. Cell Sci. *117*, 5937–5947.

Maddox, P.S., Corbett, K.D., and Desai, A. (2012). Structure, assembly and reading of centromeric chromatin. Curr. Opin. Genet. Dev. *22*, 139–147.

McWilliam, H., Li, W., Uludag, M., Squizzato, S., Park, Y.M., Buso, N., Cowley, A.P., and Lopez, R. (2013). Analysis Tool Web Services from the EMBL-EBI. Nucleic Acids Res. *41* (Web Server issue), W597–W600.

Meraldi, P., McAinsh, A.D., Rheinbay, E., and Sorger, P.K. (2006). Phylogenetic and structural analysis of centromeric DNA and kinetochore proteins. Genome Biol. *7*, R23.

Müller, S., and Almouzni, G. (2013). A network of players in H3 histone variant deposition and maintenance at centromeres. Biochim. Biophys. Acta. http://dx.doi.org/10.1016/j.bbagrm.2013.11.008.

Nechemia-Arbely, Y., Fachinetti, D., and Cleveland, D.W. (2012). Replicating centromeric chromatin: spatial and temporal control of CENP-A assembly. Exp. Cell Res. *318*, 1353–1360.

Nishino, M., Choy, J.W., Gushwa, N.N., Oses-Prieto, J.A., Koupparis, K., Burlingame, A.L., Renslo, A.R., McKerrow, J.H., and Taunton, J. (2013). Hypothemycin, a fungal natural product, identifies therapeutic targets in *Trypanosoma brucei*. Elife *2*, e00712.

Obado, S.O., Taylor, M.C., Wilkinson, S.R., Bromley, E.V., and Kelly, J.M. (2005). Functional mapping of a trypanosome centromere by chromosome fragmentation identifies a 16-kb GC-rich transcriptional "strand-switch" domain as a major feature. Genome Res. *15*, 36–43.

Obado, S.O., Bot, C., Nilsson, D., Andersson, B., and Kelly, J.M. (2007). Repetitive DNA is associated with centromeric domains in *Trypanosoma brucei* but not *Trypanosoma cruzi*. Genome Biol. *8*, R37.

Ogbadoyi, E., Ersfeld, K., Robinson, D., Sherwin, T., and Gull, K. (2000). Architecture of the *Trypanosoma brucei* nucleus during interphase and mitosis. Chromosoma *108*, 501–513.

Parsons, M., Worthey, E.A., Ward, P.N., and Mottram, J.C. (2005). Comparative analysis of the kinomes of three pathogenic trypanosomatids: *Leishmania major, Trypanosoma brucei* and *Trypanosoma cruzi*. BMC Genomics *6*, 127.

Ploubidou, A., Robinson, D.R., Docherty, R.C., Ogbadoyi, E.O., and Gull, K. (1999). Evidence for novel cell cycle checkpoints in trypanosomes: kinetoplast segregation and cytokinesis in the absence of mitosis. J. Cell Sci. *112*, 4641–4650.

Poon, S.K., Peacock, L., Gibson, W., Gull, K., and Kelly, S. (2012). A modular and optimized single marker system for generating *Trypanosoma brucei* cell lines expressing T7 RNA polymerase and the tetracycline repressor. Open Biol. *2*, 110037.

Punta, M., Coggill, P.C., Eberhardt, R.Y., Mistry, J., Tate, J., Boursnell, C., Pang, N., Forslund, K., Ceric, G., Clements, J., et al. (2012). The Pfam protein families database. Nucleic Acids Res. *40* (Database issue), D290–D301.

Quinlan, A.R., and Hall, I.M. (2010). BEDTools: a flexible suite of utilities for comparing genomic features. Bioinformatics *26*, 841–842.

Reinhardt, H.C., and Yaffe, M.B. (2013). Phospho-Ser/Thr-binding domains: navigating the cell cycle and DNA damage response. Nat. Rev. Mol. Cell Biol. *14*, 563–580.

Rogozin, I.B., Basu, M.K., Csürös, M., and Koonin, E.V. (2009). Analysis of rare genomic changes does not support the unikont-bikont phylogeny and suggests cyanobacterial symbiosis as the point of primary radiation of eukaryotes. Genome Biol. Evol. *1*, 99–113.

Santaguida, S., and Musacchio, A. (2009). The life and miracles of kinetochores. EMBO J. *28*, 2511–2531.

Schneider, C.A., Rasband, W.S., and Eliceiri, K.W. (2012). NIH Image to ImageJ: 25 years of image analysis. Nat. Methods *9*, 671–675.

Siegel, T.N., Hekstra, D.R., and Cross, G.A.M. (2008). Analysis of the *Trypanosoma brucei* cell cycle by quantitative DAPI imaging. Mol. Biochem. Parasitol. *160*, 171–174.

Siegel, T.N., Hekstra, D.R., Kemp, L.E., Figueiredo, L.M., Lowell, J.E., Fenyo, D., Wang, X., Dewell, S., and Cross, G.A.M. (2009). Four histone variants mark the boundaries of polycistronic transcription units in *Trypanosoma brucei*. Genes Dev. *23*, 1063–1076.

Sloof, P., Menke, H.H., Caspers, M.P., and Borst, P. (1983). Size fractionation of *Trypanosoma brucei* DNA: localization of the 177-bp repeat satellite DNA and a variant surface glycoprotein gene in a mini-chromosomal DNA fraction. Nucleic Acids Res. *11*, 3889–3901.

Stechmann, A., and Cavalier-Smith, T. (2002). Rooting the eukaryote tree by using a derived gene fusion. Science *297*, 89–91.

Stuart, K., Brun, R., Croft, S., Fairlamb, A., Gürtler, R.E., McKerrow, J., Reed, S., and Tarleton, R. (2008). Kinetoplastids: related protozoan pathogens, different diseases. J. Clin. Invest. *118*, 1301–1310.

Suzuki, M. (1989). SPKK, a new nucleic acid-binding unit of protein found in histone. EMBO J. *8*, 797–804.

Talbert, P.B., Bayes, J.J., and Henikoff, S. (2009). Evolution of centromeres and kinetochores: a two-part fugue. In The Kinetochore, P. De Wulf and W.C. Earnshaw, eds. (New York: Springer), pp. 1–37.

Trudgian, D.C., Thomas, B., McGowan, S.J., Kessler, B.M., Salek, M., and Acuto, O. (2010). CPFP: a central proteomics facilities pipeline. Bioinformatics *26*, 1131–1132.

Tschudi, C., Shi, H., Franklin, J.B., and Ullu, E. (2012). Small interfering RNA-producing loci in the ancient parasitic eukaryote *Trypanosoma brucei*. BMC Genomics *13*, 427.

Unnikrishnan, A., Akiyoshi, B., Biggins, S., and Tsukiyama, T. (2012). An efficient purification system for native minichromosome from *Saccharomyces cerevisiae*. Methods Mol. Biol. *833*, 115–123.

Walker, G., Dorrell, R.G., Schlacht, A., and Dacks, J.B. (2011). Eukaryotic systematics: a user's guide for cell biologists and parasitologists. Parasitology *138*, 1638–1663.

Waterhouse, A.M., Procter, J.B., Martin, D.M.A., Clamp, M., and Barton, G.J. (2009). Jalview Version 2—a multiple sequence alignment editor and analysis workbench. Bioinformatics *25*, 1189–1191.

Westermann, S., and Schleiffer, A. (2013). Family matters: structural and functional conservation of centromere-associated proteins from yeast to humans. Trends Cell Biol. *23*, 260–269.

Westhorpe, F.G., and Straight, A.F. (2013). Functions of the centromere and kinetochore in chromosome segregation. Curr. Opin. Cell Biol. *25*, 334–340.

Wickstead, B., and Gull, K. (2011). The evolution of the cytoskeleton. J. Cell Biol. *194*, 513–525.

Wickstead, B., Ersfeld, K., and Gull, K. (2002). Targeting of a tetracycline-inducible expression system to the transcriptionally silent minichromosomes of *Trypanosoma brucei*. Mol. Biochem. Parasitol. *125*, 211–216.

Wickstead, B., Ersfeld, K., and Gull, K. (2003). The mitotic stability of the mini-chromosomes of *Trypanosoma brucei*. Mol. Biochem. Parasitol. *132*, 97–100.

Wickstead, B., Ersfeld, K., and Gull, K. (2004). The small chromosomes of *Trypanosoma brucei* involved in antigenic variation are constructed around repetitive palindromes. Genome Res. *14*, 1014–1024.

Woodward, R., and Gull, K. (1990). Timing of nuclear and kinetoplast DNA replication and early morphological events in the cell cycle of *Trypanosoma brucei*. J. Cell Sci. *95*, 49–57.

Supplemental Information

EXTENDED EXPERIMENTAL PROCEDURES

Plasmid Construction

YFP-tagging constructs were made using pEnT5-Y as described previously (Kelly et al., 2007) with DNA Strider (Marck, 1988) (version 1.5 kindly provided by Christian Marck). Note that these constructs also add a TY tag to facilitate the recognition by the BB2 antibodies (Bastin et al., 1996). For N-terminal TY-YFP tagging, a ~250 bp fragment containing N-terminal end of the CDS of interest was amplified with primers that contained engineered XbaI and NotI restriction sites, and a ~250 bp fragment containing 5′UTR of interest was amplified with primers that contained engineered NotI and BamHI restriction sites. These two fragments were cloned together into the XbaI-BamHI sites downstream of TY-YFP in pEnT5-Y. Resulting plasmids contain a unique NotI site that is used for linearization and integration into the endogenous locus of interest. We could not obtain a PCR fragment for KKT6 and instead performed C-terminal tagging using the SpeI-HindIII sites upstream of YFP-TY. The tdTomato-tagging construct (pBA217 for KKT13) was made using the pBA148 vector that replaces the YFP of pEnT6B-Y (Kelly et al., 2007) with tdTomato. Inducible RNAi constructs were made using the p2T7-177 vector (Wickstead et al., 2002) by the introduction of a gene fragment (~500 bp) designed using the RNAit software to avoid off-target effects (Redmond et al., 2003). All primers used in this study are listed in Table S4.

SUPPLEMENTAL REFERENCES

Cheeseman, I.M., Chappie, J.S., Wilson-Kubalek, E.M., and Desai, A. (2006). The conserved KMN network constitutes the core microtubule-binding site of the kinetochore. Cell *127*, 983–997.

Dubin, M., Fuchs, J., Gräf, R., Schubert, I., and Nellen, W. (2010). Dynamics of a novel centromeric histone variant CenH3 reveals the evolutionary ancestral timing of centromere biogenesis. Nucleic Acids Res. *38*, 7526–7537.

Marck, C. (1988). 'DNA Strider': a 'C' program for the fast analysis of DNA and protein sequences on the Apple Macintosh family of computers. Nucleic Acids Res. *16*, 1829–1836.

Redmond, S., Vadivelu, J., and Field, M.C. (2003). RNAit: an automated web-based tool for the selection of RNAi targets in *Trypanosoma brucei*. Mol. Biochem. Parasitol. *128*, 115–118.

Schittenhelm, R.B., Heeger, S., Althoff, F., Walter, A., Heidmann, S., Mechtler, K., and Lehner, C.F. (2007). Spatial organization of a ubiquitous eukaryotic kinetochore protein network in *Drosophila* chromosomes. Chromosoma *116*, 385–402.

Zubácová, Z., Hostomská, J., and Tachezy, J. (2012). Histone H3 Variants in *Trichomonas vaginalis*. Eukaryot. Cell *11*, 654–661.

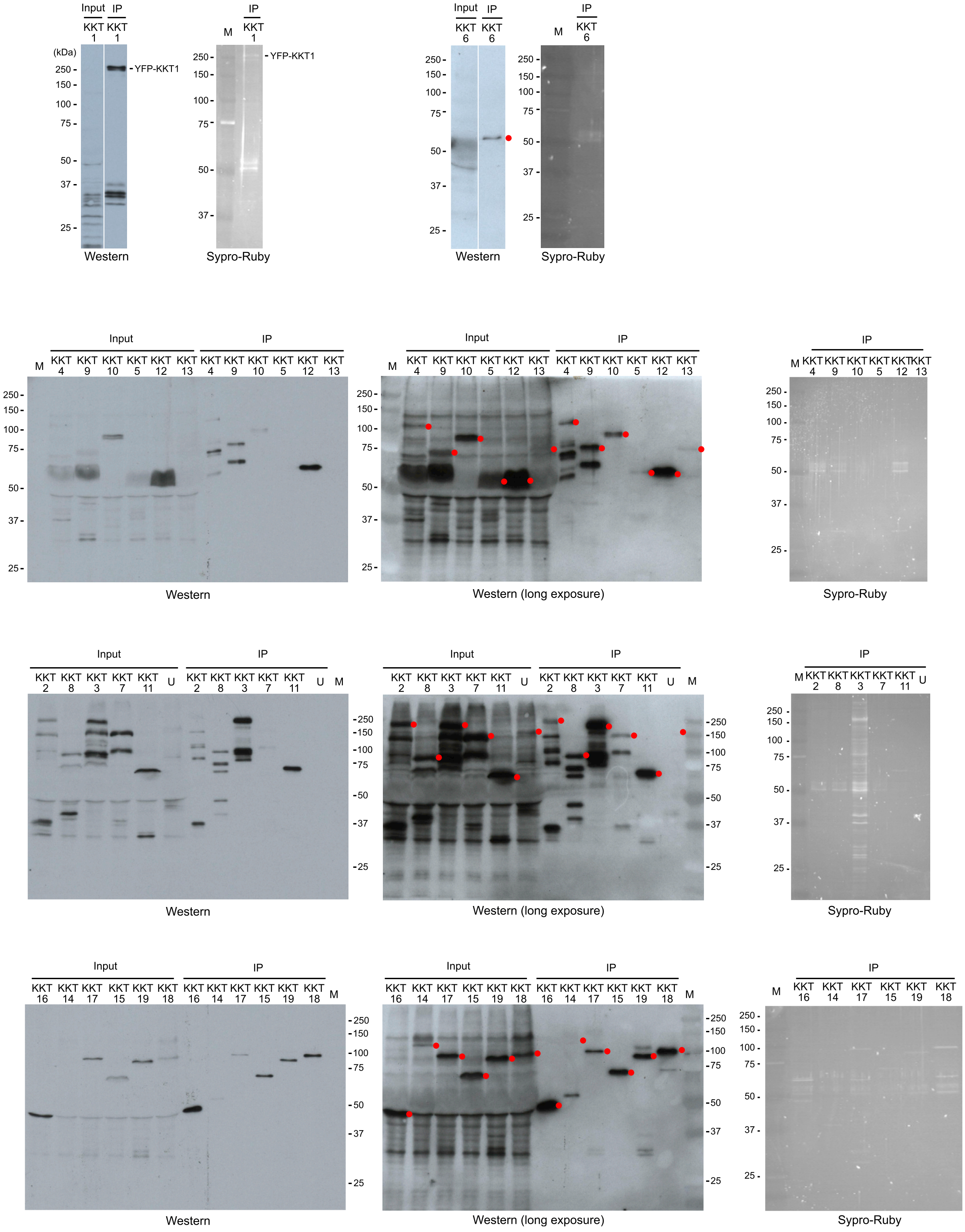

Figure S1. Affinity Purification of KKT Proteins, Related to Figure 1

YFP-tagged versions of indicated KKT proteins were purified using anti-GFP antibodies, eluted with detergent and analyzed by SDS-PAGE followed by immunoblots or Sypro-Ruby staining. "U" is a purification of an unrelated protein. Red dots indicate epitope-tagged proteins. Note that the amounts of purified proteins were often below the detection limit of Sypro-Ruby stain, yet they were identified by mass spectrometry (see Table S2).

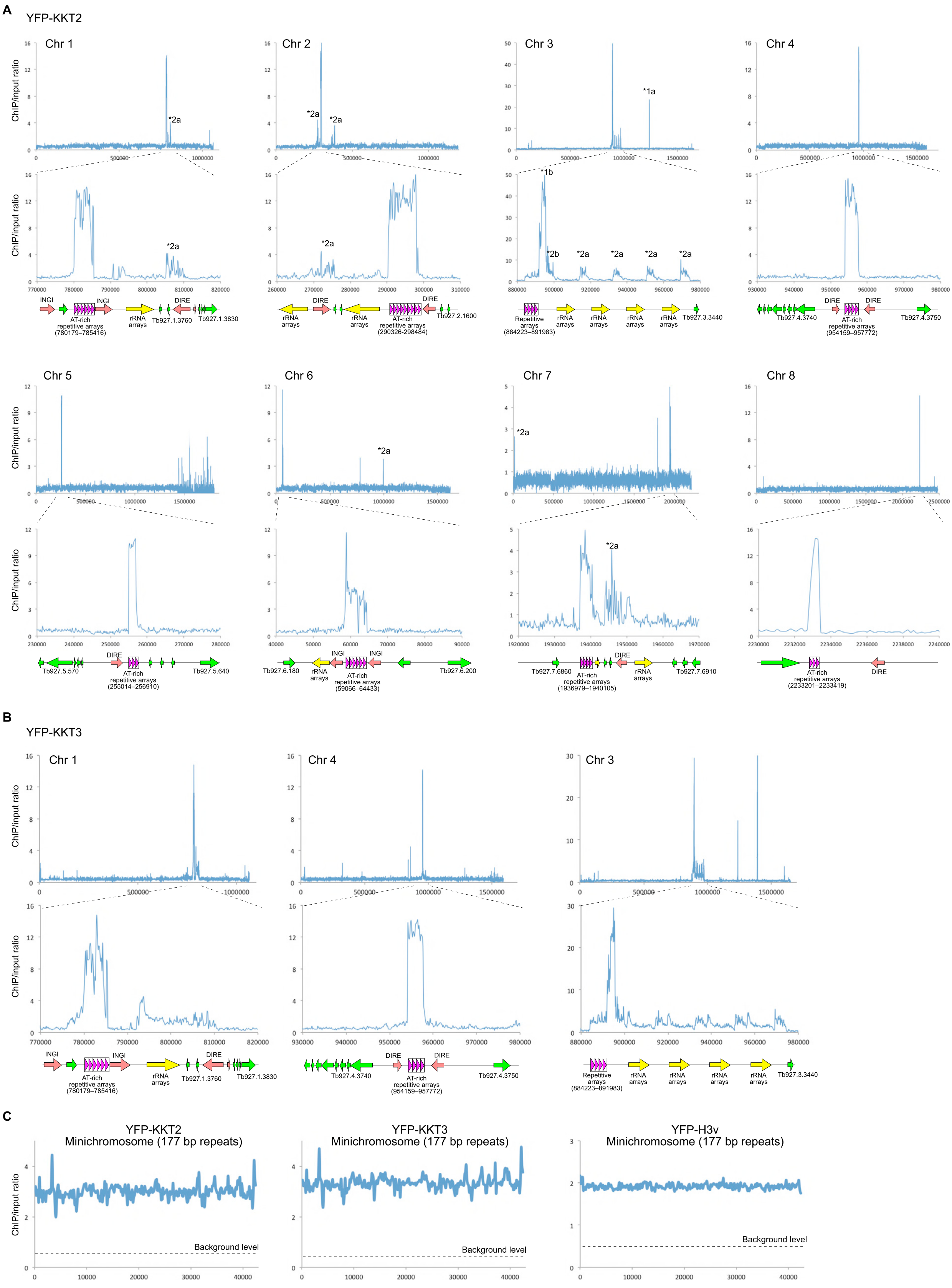

(legend on next page)

Figure S2. More ChIP-Seq Data, Related to Figure 2

(A) ChIP-seq data of YFP-KKT2 for chromosomes 1–8 (data for chromosomes 1, 3 and 4 are taken from Figure 2A). Some of the non-centromeric peaks likely represent false-positive signals due to the presence of identical sequences within the centromeres. For example, the *1a peak (1,236,842–1,238,050, which is located in between two polycistronic transcription units) is likely false-positive due to the presence of an identical sequence in the centromere of chromosome 3 (*1b: 892,214–893,422). Similarly, the *2a peaks found around some DIRE sequences may be caused by an identical sequence in the centromere of chromosome 3 (*2b).

(B) ChIP-seq data of YFP-KKT3 for chromosomes 1, 3 and 4.

(C) ChIP-seq data of YFP-KKT2, YFP-KKT3 and YFP-H3v for a model minichromosome that mostly consists of 177 bp repeats. The background level for each protein is indicated by dotted lines.

Cell

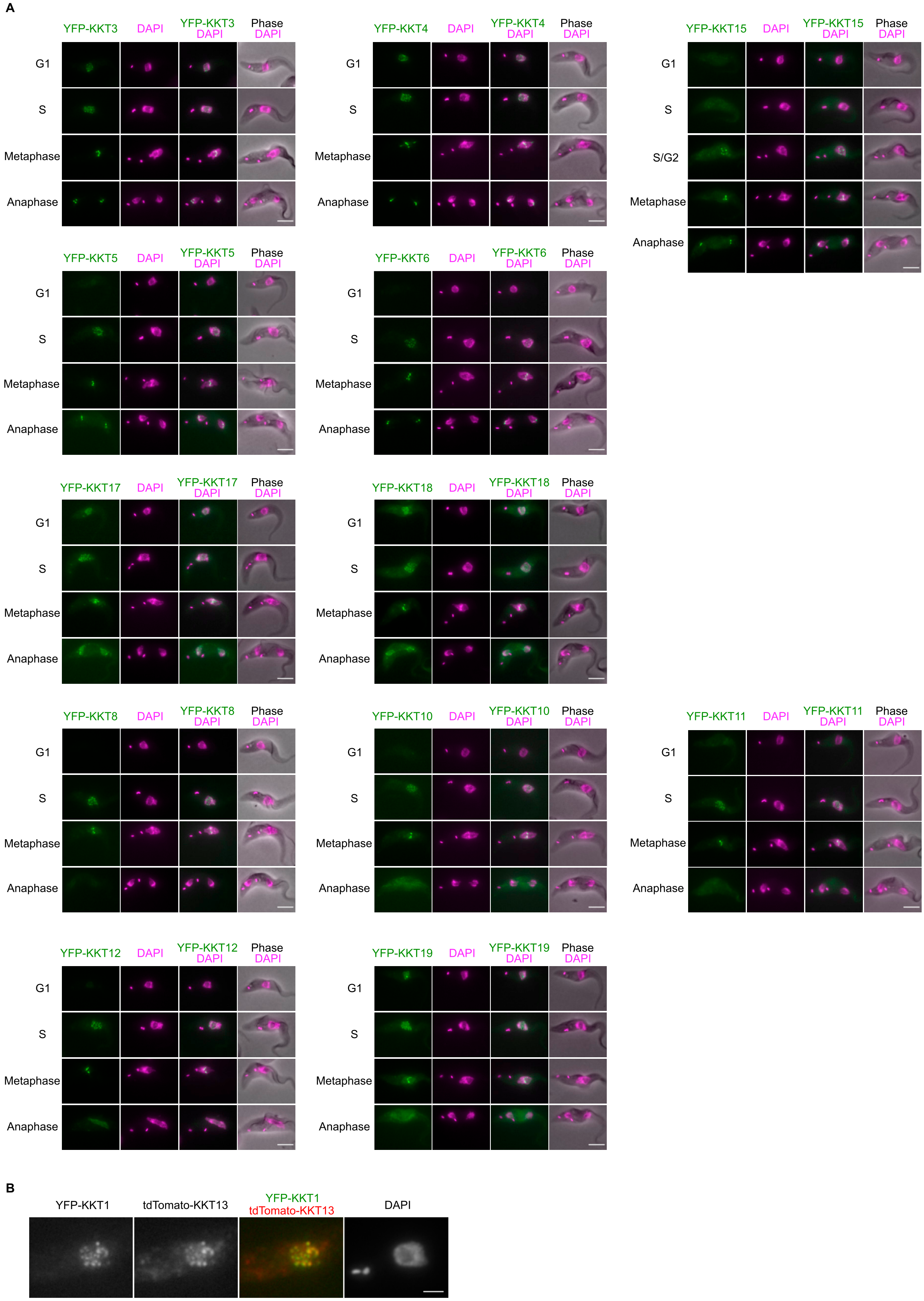

(legend on next page)

Cell

Figure S3. KKT Proteins Show Differential Localization Timings, Related to Figure 4

(A) This figure contains localization data for YFP-tagged KKT proteins not shown in Figures 1 or 4. Note that KKT4 has spindle-like signals besides kinetochore dots during metaphase. Bars, 5 μm.

(B) tdTomato-KKT13 co-localizes with YFP-KKT1, confirming that S-phase-specific KKT13 is indeed a kinetochore protein. Bar, 2 μm.

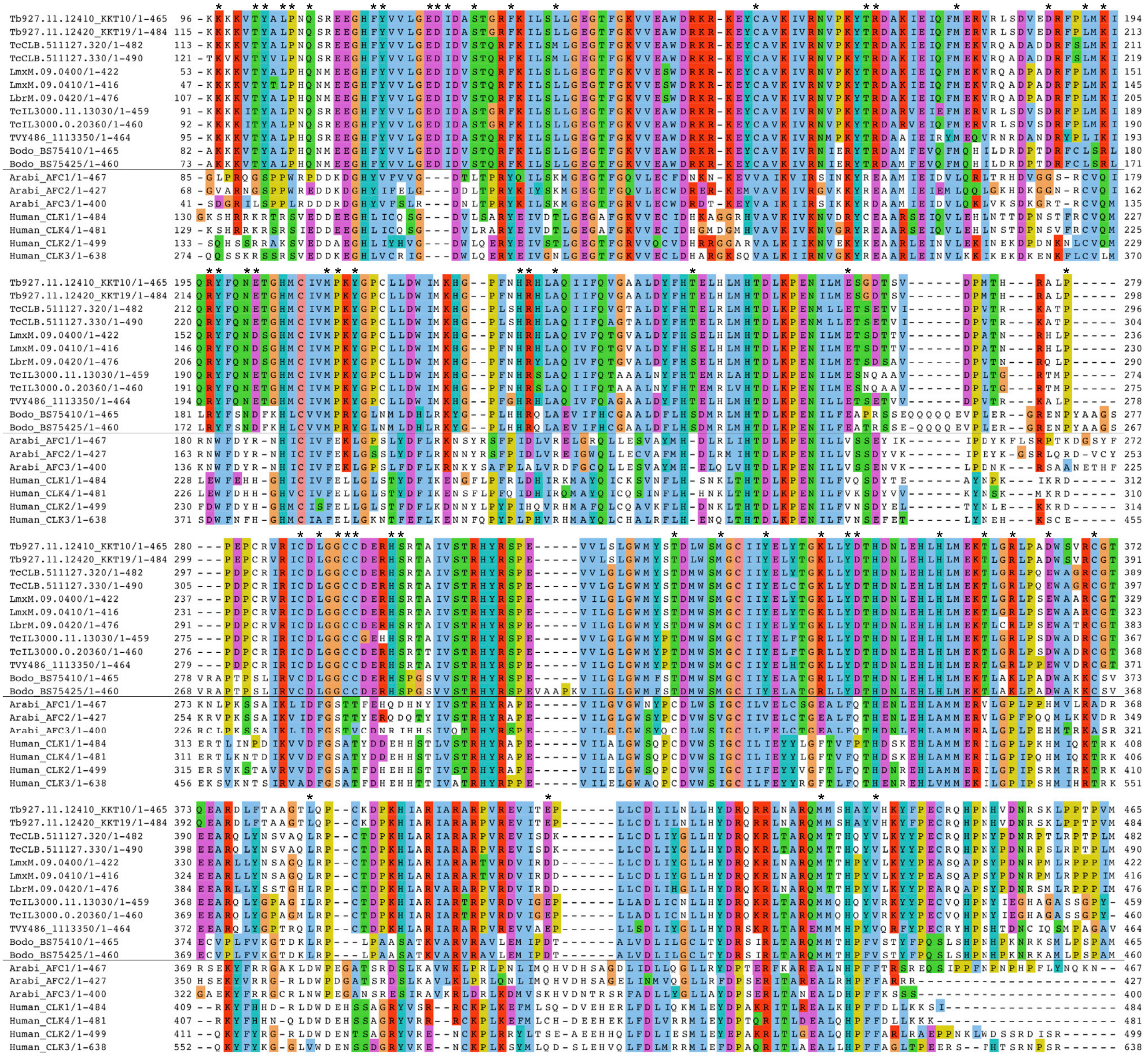

Figure S4. KKT10 and KKT19 Have Significant Differences from Other CLKs, Related to Figure 5

A multiple sequence alignment of kinetoplastid KKT10/KKT19 proteins with human and *Arabidopsis* CLKs is shown. Asterisks indicate residues that are well conserved among kinetoplastids but not in human or *Arabidopsis*.

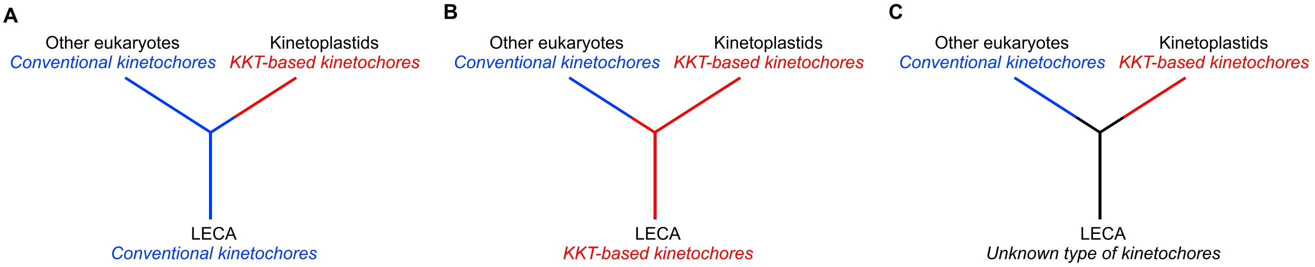

Figure S5. Three Possible Models for the Evolutionary History of Kinetochores, Related to Figure 5

(A) The LECA (last eukaryotic common ancestor) had conventional kinetochores. In this scenario, kinetoplastids lost conventional kinetochores and evolved KKT-based kinetochores after they branched from other eukaryotic lineages.

(B) The LECA had KKT-based kinetochores. In this scenario, only kinetoplastids retained them.

(C) The LECA had a hitherto unknown type of kinetochores.

Note that this is a highly simplified set of possibilities of how the diversity of kinetochore types may have arisen in evolution. The diagrams are presented as simple branch points and do not incorporate multiple other branch points leading to the diversity of other eukaryotic groups.
